# Knockdown of DNA polymerase ζ relieved the chemoresistance of glioma via inhibiting the PI3K/AKT signaling pathway

**DOI:** 10.1080/21655979.2021.1944027

**Published:** 2021-07-19

**Authors:** Junbao Yang, Weilong Ding, Xiangyu Wang, Yongsheng Xiang

**Affiliations:** Department of Neurosurgery, The First Affiliated Hospital of Jinan University, Guangzhou, Guangdong, China

**Keywords:** DNA polymerase ζ, glioma, REV7, chemoresistance

## Abstract

Previous reports suggest that DNA polymerase ζ is highly expressed in glioma tissues. The present study aimed to investigate the roles of the REV7 subunit of DNA polymerase ζ in glioma cell chemoresistance and its underlying mechanisms. The bioinformatics method was used to compare the expression of REV7 in glioma and normal tissues. The expression of REV7 in glioma tumor samples and the adjacent tissue was examined by reverse transcription polymerase chain reaction. Moreover, an in vitro analysis using glioma cells was used to test the effects of REV7 siRNA on the proliferation and apoptosis of glioma cell line U251 cells, and the effect of REV7 siRNA on the sensitivity of the U251 cells to cisplatin was also explored. The expression of REV7 in glioma tumors was significantly increased. Moreover, the knockdown of REV7 in glioma cells decreased the proliferation and increased the apoptosis of U251 cells; moreover, REV7 siRNA also increased the sensitivity of U251 cells to cisplatin. Finally, REV7 may regulate the proliferation, apoptosis, and chemosensitivity of U251 cells by affecting phosphoinositide 3-kinase signaling. Our data suggest that REV7 is involved in the chemosensitivity of glioma cells and provides a theoretical basis for targeting DNA polymerase ζ to improve the sensitivity of glioma cells to chemotherapy.

## Graphical Abstract

**Figure:**
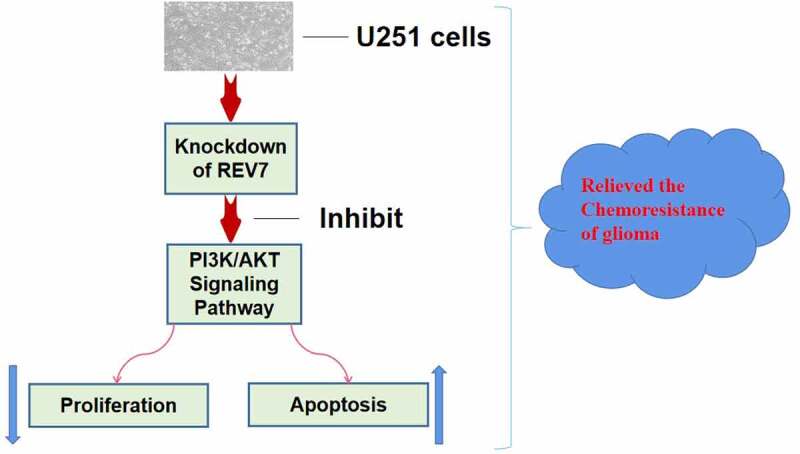

## Introduction

In recent years, glioma has become one of the most commonly diagnosed types of cancer worldwide [1–3]. With the recurrence and metastasis ability of cancer cells, the number of patients diagnosed with malignant glioma is increasing [3–5]. Current anti-glioma therapies include chemotherapy and surgery, and previous research has shown that chemotherapy can improve the lifespan and quality of life of most glioma patients [6,7]. Nevertheless, the resistance of glioma cells to chemotherapy is a key factor affecting the efficacy of chemotherapy. Therefore, it is important to identify the mechanism underlying chemotherapy resistance and develop effective methods to improve the chemosensitivity of glioma cells.

The DNA repair mechanism of tumor cells is a key factor affecting cell chemosensitivity. Recently, studies have shown that translesion DNA synthesis (TLS) is an emergency repair mechanism in eukaryotic cells [8–10]. Translesion DNA synthesis plays an important role in the repair of DNA damage induced by ionizing radiation, and therefore enhances the radiation resistance of tumor cells. DNA polymerase ζ (pol ζ) is the key component of TLS [9–12]. It mainly consists of two subunits, REV3L and REV7. The REV3L protein is the catalytic subunit and REV7 is the structural subunit [11]. Several studies have shown that the subunits of pol ζ are highly expressed in gliomas and are associated with patient survival [13–15]. However, the molecular mechanisms and signaling pathways involved in the process of radiation resistance induced by pol ζ require further investigation.

In this research, we aimed to explore the roles of the REV7 subunit of polymerase ζ in the chemosensitivity of glioma cells as well as its underlying mechanism. We hypothesized that knockdown of REV7 can regulate the chemosensitivity of glioma cells via inhibiting the PI3K/AKT signaling pathway.

## Materials and methods

### Clinical samples

Glioma tumor tissues and paired adjacent normal tissues were collected from 40 glioma patients who underwent surgery at the Department of Neurosurgery, The First Affiliated Hospital of Jinan University, between August 2018 and September 2020. The clinical characteristic of the glioma patients was shown in Table 1. All tissue samples were collected, frozen in liquid nitrogen, and stored at −80°C for further use.

**Table 1.** Clinical characteristic of the glioma patients

	REV7 low group (n = 17)	REV7 high group (n = 23)	P value
Age			0.9733
≥60	11	15	
<60	6	8	
Gender			0.6161
Male	9	14	
Female	8	9	
Tumor size			0.0313*
≥5	6	16	
<5	11	7	
WHO grade			0.3373
I–II	7	13	
III–IV	10	10	
Metastasis			0.2508
Yes	8	15	
No	9	8	

*p < 0.05

The present study was approved by the ethical committee of The First Affiliated Hospital of Jinan University, and informed consent was obtained from each patient.

### Cell culture and transfection

The human glioma cell line U251 was obtained from the American Type Culture Collection (ATCC). Cells were cultured in DMEM (Invitrogen) supplemented with 10% fetal bovine serum (Invitrogen) in a humidified incubator at 37°C and 5% CO_2_. The cells were treated with cisplatin at different doses. To knockdown REV7, two siRNAs were synthesized (GenePharma) and co-transfected into glioma cells using Lipofectamine 3000 (Invitrogen) following the manufacturer’s instructions.

### RNA extraction and reverse transcription polymerase chain reaction analysis

The glioma tissue samples and U251 cells were collected, and RNA was extracted using TRIzol (Invitrogen). Quantitative reverse transcription polymerase chain reaction (qRT-PCR) was performed using the One-Step TB Green® PrimeScript™ RT-PCR Kit (TaKaRa). The ABI 7500 Real-Time PCR System (Applied Biosystems) was used for the PCR reactions. The reaction conditions were: 95°C for 30s, followed by 40 cycles of 95°C for 5 s, 60°C for 30 s, and dissociation at 95°C for 15 s, 60°C for 30 s, and 95°C for 15 s. All procedures strictly followed the manufacturer’s instructions. The primers were synthesized by Sangon Biotech (Shanghai, China). Gene expression was normalized to the expression of glyceraldehyde 3-phosphate dehydrogenase as an internal control. Relative gene expression was calculated by the 2^−ΔΔCt^ method as previous study described [16].

### Western blot

The western blot was performed accroding to Zhao et al. [17]. Glioma tissue samples and U251 cells were lysed using radio immunoprecipitation solution (Beyotime) to isolate the proteins, and the protein concentration was measured using the Bradford Protein Assay Kit (Beyotime). Gel electrophoresis was then performed, and then the proteins were transferred to the polyvinylidene difluoride membrane, blocked with 5% nonfat milk, and incubated with the primary antibodies at 4°C overnight, after which the membranes were washed and incubated with horseradish peroxidase-conjugated secondary antibodies. Finally, the protein bands were visualized using an enhanced chemiluminescence kit (Beyotime) and imaged using a Tannon 6100 imaging system (Tannon, Shanghai, China).

### Cell viability assay

The cell viability was detected by CCK-8 assay accroding to the study reported by Barrios et al [18]. Briefly, cells were placed in 96‐well plates, treated with IR, and incubated with 10 μl of CCK‐8 reagent (Beyotime). The optical density was measured using a microplate reader at 450 nm.

### Colony formation assay

Colony formation assay has been used to determine the proliferation ability of the U251 cells as Wang et al. described [19]. The colony formation assay was used to determine the proliferation ability of the U251 cells. Briefly, the cells were cultured for 10 days and then stained with crystal violet (0.1%). Then, the colonies (diameter ≥ 100 μm) were counted and imaged under a microscope.

### Cell apoptosis analysis

For the apoptosis analysis, after transfection and IR treatment, the glioma cells were stained with the PI/Annexin V-FITC apoptosis kit (Beyotime). Briefly, the cells were digested with trypsin and collected. The cells were then centrifuged for 3 min. Next, 200 µl of 1× binding buffer and 10 µm Annexin V-APC staining were added. The cell apoptosis rate was analyzed using a BD FACSVerse flow cytometer (BD Bioscience) following the manufacturer’s instructions.

### Statistical analysis

Statistical analyses were performed using SPSS 19.0. The data were expressed as the mean ± standard deviation (SD). Comparisons between the two groups were performed using a Student’s t-test, and comparisons among multiple groups were analyzed by ANOVA. Statistical significance was set at p < 0.05.

## Results

In this study, we aimed to explore the effects of the REV7 subunit of polymerase ζ on the chemosensitivity of glioma cells . We hypothesized that knockdown of REV7 inhibit the proliferation and promote the apoptosis of U251 cells, and regulate the chemosensitivity of glioma cells via inhibiting the PI3K/AKT signaling pathway.

### Clinical characteristics of the patients

The clinical information of the 40 glioma patients were shown in Table 1. There is no difference in age, gender, WHO grade, and metastasis between the two groups. However, the tumor size was significantly higher in the REV7 high group compared with the REV7 low group.

### Upregulated expression of REV7 in human glioma cancer tissues

**Figure 1.**
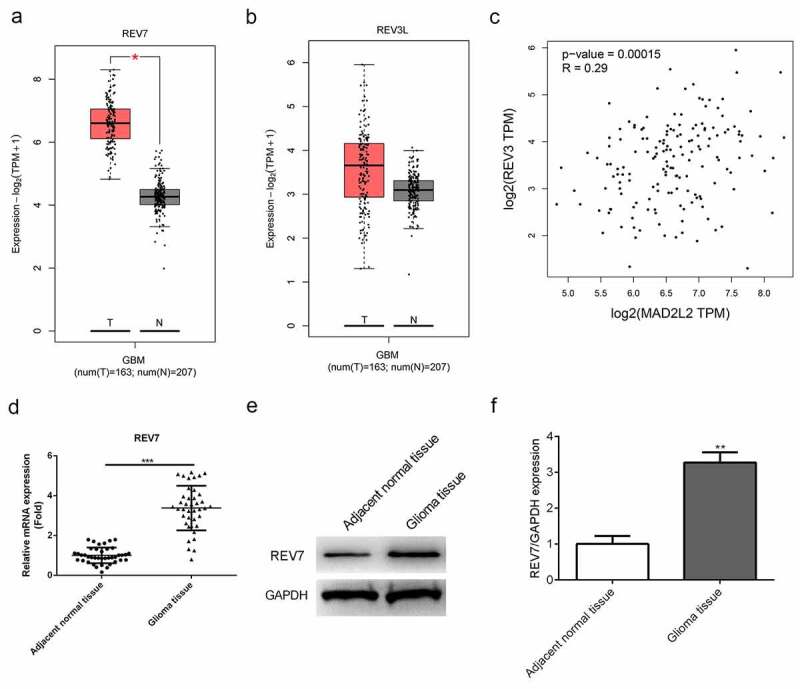
Overexpression of REV7 in glioma tissue. (a–b) The expression of REV3L and REV7 in the tumor tissues and adjacent tissues were obtained from the TCGA database. (c) The correlation analysis of REV3 and MAD2L2 was analyzed with TCGA database. (d) The mRNA expression of REV7 was measured by the quantitative reverse transcription polymerase chain reaction analysis. (e–f) The protein expression of REV7 was detected by western blot. * p < 0.05, ** p < 0.01, * p < 0.001. GBM,;T,;N,;TPM,;GAPDH, glyceraldehyde 3-phosphate dehydrogenase

First, we performed bioinformatic analysis using data from the TCGA database using the online tool GEPIA, and the expressions of the two subunits of pol ζ are shown in Figure 1. We found that the expression of REV7 (Figure 1a) was significantly increased in the glioma tissue compared with that in the normal tissue (p < 0.05); the expression of REV3L also increased in the glioma tissue, but no significant differences were observed (Figure 1b, p > 0.05). Moreover, the results of the correlation analysis showed that the levels of REV7 and REV3L were positively correlated in glioma tissues (Figure 1c, r = 0.29, p = 0.00015); furthermore, the mRNA and protein expression of REV7 in glioma tissues were determined by the qRT-PCR and western blot methods, respectively. As shown in Figure 1d, the mRNA expression of REV7 was obviously increased in glioma tissues compared with that in non-tumor tissues (p < 0.01). The protein expression of REV7 was also significantly upregulated in glioma tissues (p < 0.01, Figure 1e).

### Knockdown of REV7 inhibited the proliferation and promoted the apoptosis of U251 cells in vitro

**Figure 2.**
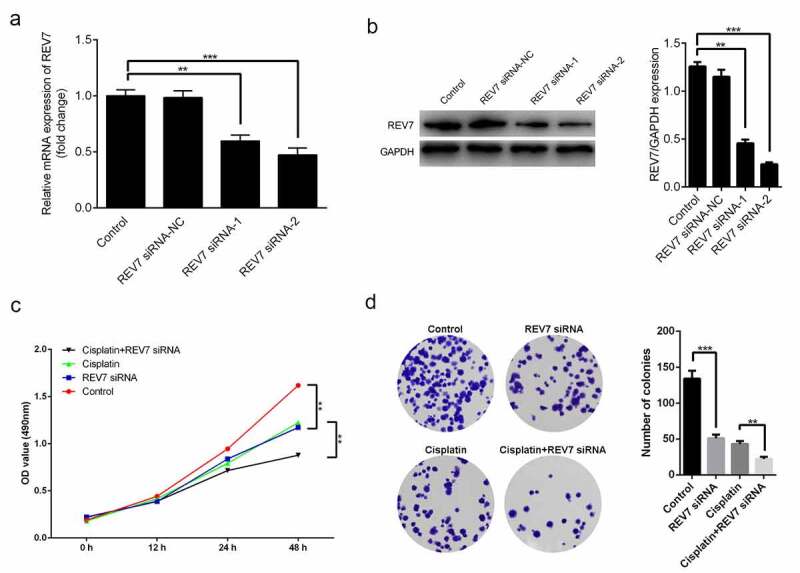
Effect of REV7 siRNA on the proliferation of U251 cells. (a) The mRNA expression of REV7 was measured by the qRT-PCR analysis after REV7 siRNA transfection. (b) The protein expression of REV7 was measured by quantitative reverse transcription polymerase chain reaction (qRT-PCR) analysis after REV7 siRNA transfection. (c) The cell viability was demonstrated by the CCK-8 assay after Cisplatin and REV7 siRNA treatment. (d) The proliferation of the cells was detected by colony formation assay after Cisplatin and REV7 siRNA treatment. ** p < 0.01, * p < 0.001. GAPDH, glyceraldehyde 3-phosphate dehydrogenase; OD, optical density

**Figure 3.**
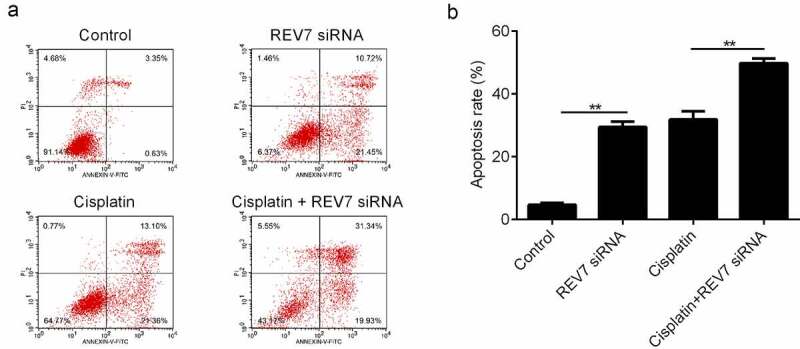
Effect of REV7 siRNA on the apoptosis of U251 cells. (a–b) The apoptosis rate was measured by flow cytometry after Cisplatin and REV7 siRNA treatment. ** p < 0.01. PI,; ANNEXIN-V-F

Next, glioma cells U251 cells were transfected with REV7 siRNA. Figure 2a, b indicate that the mRNA and protein expression levels of REV7 were significantly decreased in both the siRNA-1 and siRNA-2 groups. The siRNA-2 REV7 treatment was more effective in downregulating REV7, so it was used in downstream experiments. Moreover, the effects of REV7 siRNA on the growth and metastasis of U251 cells were measured by MTT, colony formation, and flow cytometry assays. The knockdown of REV7 markedly inhibited the proliferation of U251 cells (Figure 2c, d) and promoted apoptosis (Figure 3) *in vitro* (p < 0.01).

### Downregulation of REV7 promoted the chemosensitivity of U251 cell to cisplatin

Furthermore, the U251 cells were treated with cisplatin with or without REV7 siRNA, and the effect of REV7 siRNA on the chemosensitivity of the U251 cells was determined. Compared with the cisplatin-treated U251 cells, cell proliferation was significantly decreased and apoptosis was markedly increased in the cisplatin+REV7 siRNA group (Figures 2, 3).

### REV7 may regulate the proliferation, apoptosis, and cisplatin sensitivity of U251 cells via phosphoinositide 3-kinase (PI3K)/AKT signaling

**Figure 4.**
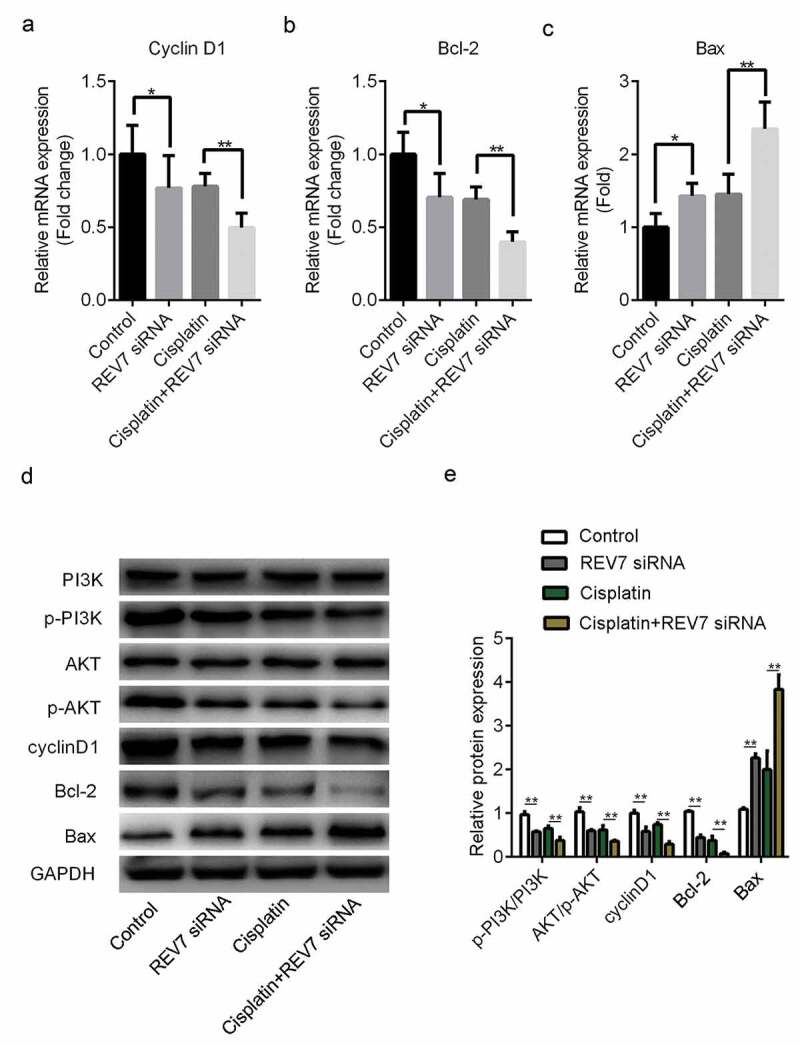
Effect of REV7 siRNA on phosphoinositide 3-kinase (PI3K)/AKT signaling in U251 cells. The mRNA expression levels of cyclin D1 (a), Bcl-2 (b), and Bax (c) were detected by reverse transcription polymerase chain reaction after cisplatin and REV7 siRNA treatment. (d–e) The protein expression levels of cyclin D1, Bcl-2, and Bax PI3K/AKT signaling pathways were detected by western blotting. * p < 0.05, ** p < 0.01. GAPDH, glyceraldehyde 3-phosphate dehydrogenase

Finally, the potential underlying mechanism of REV7 on the proliferation, apoptosis, and cisplatin sensitivity of U251 cells was evaluated. As shown in Figure 4, compared with the control group, the mRNA and/or protein expression levels of p-PI3K, p-AKT, cyclin D1, and Bcl-2 were significantly downregulated in the REV7 siRNA-treated group (p < 0.01). The expression level of Bax sharply increased in REV7 siRNA-treated U251 cells (p < 0.01). Moreover, compared with the cisplatin group, the expression levels of p-PI3K, p-AKT, cyclin D1, and Bcl-2 were significantly downregulated and the expression of Bax was increased compared with those in the cisplatin+siRNA NC group (p < 0.01).

## Discussion

Glioma is regarded as one of the dominant causes of cancer globally, and its pathological complexity necessitates combination therapies of various therapeutic elements, such as anti-cancer drugs and genes, to achieve synergistic treatment [4,5,20]. REV7 is an important subunit of pol ζ and is highly evolutionarily conserved. DNA polymerase ζ was found to play an essential role in the transcriptional regulation process and participates in many important biological processes, such as embryonic development and tumorigenesis. It has been reported that the two main functional subunits of pol ζ, REV3L and REV7, play important roles in the development of lung tumors [21,22], breast tumors [23], colorectal tumors [24], and cervical tumors [25]. Both REV3L and REV7 were reported to regulate cell proliferation, apoptosis, cycle, invasion, and angiogenesis, and their abnormal regulation and functioning are closely related to tumor development. Therefore, pol ζ is expected to be a potential target for cancer treatment.

The role of pol ζ in gliomas has also been discussed [13,14]. In the current study, we performed a bioinformatic analysis and, based on the data from the TCGA database, both REV3L and REV7 were found to be upregulated in glioma tissues, with the levels of both REV3L and REV7 positively correlated. Moreover, the results of the qRT-PCR analysis showed that REV7 was significantly upregulated in glioma tissue compared with the adjacent normal tissue, which was consistent with the results of previous studies as well as the bioinformatic analysis. Moreover, the results of cell experiments showed that knockdown of REV7 in U251 cells inhibited U251 cell proliferation and increased cell apoptosis in vitro. Taken together, these results suggest that REV7 is upregulated in gliomas and functions as an oncogene.

Cisplatin is a commonly used chemotherapeutic agent for the treatment of many cancers [26–28]. However, the development of cisplatin resistance is one of the main reasons for low chemotherapeutic efficiency in patients with tumors [21,26]. Therefore, improving the treatment strategies for cisplatin-resistant glioma may improve its clinical application. A previous study discussed the role of REV3L in regulating the chemosensitivity of glioma cells, suggesting that pol ζ is involved in the drug resistance of glioma [14]. However, the role of REV7 in the chemosensitivity of gliomas has not yet been discussed. In the present study, we explored the effect of REV7 on the sensitivity of glioma cells to cisplatin. Cell proliferation was markedly suppressed in the cisplatin-treated group compared with that in the control group, while cell apoptosis was significantly increased. More importantly, with the treatment of siRNA REV7, the cell proliferation of the cisplatin+siRNA REV7 group decreased significantly compared with the cisplatin administration alone. To further verify these results, we measured the expressions of Cyclin D1, Bax and Bcl-2. Cyclin D1 is a nuclear factor of cell cycle, which can induce cell proliferation [29]; Bcl-2, as a member of the Bcl family, inhibited the cell apoptosis. While the trend of Bax is opposite to that of Bcl-2, which is a kind of a pro apoptotic factor [30]. In this study, we confirmed that knockdown of REV7 further down-regulated the expressions of Cyclin D1 and Bcl-2, and up-regulated the Bax expression. These results further demonstrated that REV7 re-sensitized the glioma cells to cisplatin probably by inhibiting cell proliferation, inducing apoptosis.

Mutations in the PI3K/AKT pathway are frequently found in cancers and are associated with cellular transformation, carcinogenesis, tumor progression, and drug resistance [1,31]. The activation of AKT contributes to carcinogenesis, tumor metastasis, and, as shown most recently, resistance to chemotherapy [32,33]. Modulation of AKT activity is now a commonly observed endpoint of chemotherapy administration. Studies that combined small-molecule inhibitors of the PI3K/AKT pathway with standard chemotherapy have been successful in attenuating chemotherapeutic resistance. Specifically, inhibiting AKT activity may be a valid approach to treat cancer and increase the efficacy of chemotherapy. In this study, the ratio of p-PI3K/PI3K and p-AKT/AKT was significantly decreased in the REV7 siRNA-treated group. Moreover, the knockdown of REV7 also led to changes in the downstream molecules. Meanwhile, the expression levels of the PI3K/AKT signaling molecules after treatment with siRNA REV7 were significantly reduced compared with the cisplatin-treated group. Taken together, the above results suggest that REV7 may affect the growth, apoptosis, and chemosensitivity of U251 cells by regulating PI3K/AKT signaling.

However, there are still some restrictions on our study. In the future, we need collect more clinical samples to analyze the REV7 expression, and explore the role of REV7 by establishing xenograft animal models.

## Conclusion

Altogether, this study suggests that REV7 is increased in glioma tissues and REV7 sensitized cisplatin via the modulation of the PI3K/AKT signaling pathway, thereby mediating cell proliferation and apoptosis in vitro. These results can be used for the development of novel diagnostic therapeutic strategies and may suggest potential therapeutic targets for glioma tumors.

## Highlights

- REV7 is significantly higher in glioma tissues than surrounding tissues
- REV7 knockdown in glioma tissues decreased cell proliferation and increased apoptosis
- REV7 siRNA increased glioma chemosensitivity to cisplatin

REV7 regulates glioma proliferation, apoptosis, and chemosensitivity by PI3K pathway.
